# CT-guided iodine-125 seed permanent implantation for recurrent head and neck cancers

**DOI:** 10.1186/1748-717X-5-68

**Published:** 2010-07-30

**Authors:** Yu L Jiang, Na Meng, Jun J Wang, Ping Jiang, Hui SH Yuan, Chen Liu, Ang Qu, Rui J Yang

**Affiliations:** 1 Department of Radiation Oncology, Peking University Third Hospital, Beijing 100191, PR China; 2 Department of Radiology, Peking University Third Hospital, Beijing, 100191, PR China

## Abstract

**Background:**

To investigate the feasibility, and safety of ^125^I seed permanent implantation for recurrent head and neck carcinoma under CT-guidance.

**Results:**

A retrospective study on 14 patients with recurrent head and neck cancers undergone ^125^I seed implantation with different seed activities. The post-plan showed that the actuarial D90 of ^125^I seeds ranged from 90 to 218 Gy (median, 157.5 Gy). The follow-up was 3 to 60 months (median, 13 months). The median local control was 18 months (95% CI, 6.1-29.9 months), and the 1-, 2-, 3-, and 5- year local controls were 52%, 39%, 39%, and 39%, respectively. The 1-, 2-, 3-, and 5- survival rates were 65%, 39%, 39% and 39%, respectively, with a median survival time of 20 months (95% CI, 8.7-31.3 months). Of all patients, 28.6% (4/14) died of local recurrence, 7.1% (1/14) died of metastases, one patient died of hepatocirrhosis, and 8 patients are still alive to the date of data analysis.

**Conclusion:**

CT-guided ^125^I seed implantation is feasible and safe as a salvage or palliative treatment for patients with recurrent head and neck cancers.

## Background

Most patients who have ever undergone surgery for head and neck cancer or those local advanced or regional recurrence cancer patients received surgery combined with adjuvant external-beam radiotherapy (EBRT) [1,2]. Management of patients with recurrent head and neck cancers after surgery, EBRT, and adjuvant chemotherapy is a challenge for clinical oncologists. Salvage surgery is often technically feasible after the patients treated with full doses of EBRT, but the curative potential of surgery alone is low; further, the morbidity is high [3]. Redelivery of effective doses of EBRT is difficult because of the limited tolerance of adjacent normal tissues. Therefore, there has been a growing interest in combining salvage surgery with intraoperative interstitial brachytherapy [4].

Temporary intraoperative interstitial brachytherapy has its advantage in this aspect, in which a high dose of radiation is delivered directly to the cancer, while ensuring that a much lower dose is delivered to the adjacent normal structures [5-7]. The use of high-dose rate (HDR) and low-dose rate (LDR) brachytherapy in previously irradiated regions is often safe because the use of implants ensures the delivery of the dose to very specific and limited volumes of tissue. A variety of isotopes are available for use as HDR or LDR temporary interstitial brachytherapy, but the most commonly used isotope is Iridium-192, Co-60 or Cs-137 et al, especially for treating recurrent solid cancers [8-10]. However, the use of those isotopes for intraoperative HDR brachytherapy requires very complicated shielding [11].

Computed tomography (CT)-guided permanent brachytherapy was initially used for treating liver malignancies [12,13]. This novel technique ensures protracted cell killing over a period of several months through targeted delivery of high-dose radiation. The advantages of this technique are as follows: (1) it is minimally invasive, (2) dose distribution can be accurately predicted, (3) continuous irradiation increases the likelihood of damaging malignant cells in a vulnerable phase of the cell cycle, and (4) the incidence rate of acute adverse effects is low.

We have gained significant experience in using permanent ^125^I seed implantation for treating recurrent rectal cancer and spinal metastases [14,15]. The theoretical benefit of seed permanent implantation as a salvage treatment is enhanced disease control in the region of recurrence through precise and continuous LDR irradiation, which, in turn, minimizes injury to the overlying skin and surrounding neurovascular structures.

In this study, we investigated the efficacy and feasibility of percutaneous CT-guided ^125^I seed permanent implantation for recurrent head and neck cancers and analyzed the local control, survival and complications of this modality.

## Patients and methods

We conducted a retrospective analysis of 14 patients (median age, 40 years; range, 19-74 years) who had been treated for recurrent head and neck cancers with CT-guided ^125^I seed permanent implantation at Peking University Third Hospital between Feb 2003 and November 2009. The study population included 9 male and 5 female patients. The criteria for eligibility were as follows: histologically proven recurrent head and neck cancer after surgery and radiotherapy, without any evidence of distant metastasis; a Karnofsky Performance Status (KPS) score ≥60; and no severe impairment of kidney, liver, or bone marrow function. The diameter of recurrent tumor was less than 7 cm. All patients had ever been reviewed by the surgeons and radiation oncologists, and were considered not suitable for salvage surgery and EBRT again or the patients refused to receive surgery and EBRT further.

Before the operation, we evaluated the history and physical condition of all the patients, hematological and chemical tests, and obtained CT images of the head and neck and radiographs of the chest. Patient characteristics are shown in Table 1.

**Table 1:** Patient characteristics (n = 14)

	No. of patients	Percentage (%)
Median age	40(range,19-74)	
Gender		
Male	9	64
Female	5	36
KPS		
60	1	7
70	4	29
80	6	43
90	2	14
100	1	7
Primary tumor stage		
Stage I	1	7
Stage II	3	21
Stage III	4	29
Stage IV	5	36
Unclear	1	7
Primary tumor		
Sinus paranasal	5	36
Nasopharynx carcinoma	3	21
Larynx	2	14.3
Hypopharyngeal carcinoma	1	7
Nasal cavity	1	7
Mandible	1	7
Infratemporal fossa	1	7
Previous surgery	8	57
Previous chemotherapy	6	43
Previous radiotherapy	12	86
One	7	50
Two	4	29
Four	1	7
Previous cumulative dose(Gy)		
≤50 Gy	2	14
50 ~ 100 Gy	5	36
>100 Gy	5	36
Median dose(Gy)	70(range,50-250)	

Of the 14 patients, 2 had undergone radical surgery alone, 6 had received EBRT alone, and 6 had undergone surgery and EBRT. Two patients had undergone surgery twice and the other two patients had undergone surgery three times. Among the patients who underwent EBRT, 1, 5, and 6 patients received it 4 times, twice, and once, respectively. The total dose delivered to PTV ranged from 48.5 to 250 Gy (median, 70 Gy). Six patients had been administered chemotherapy (3-13 cycles; median, 4 cycles). Five patients had regional recurrence in the lymph nodes and 9 patients had recurrence in the primary lesions.

A detailed CT-aided tumor-volume study was also performed for all the patients 1-2 weeks before seed implantation. We obtained transverse images of the targets at 5-mm intervals. The images were transferred to a computerized treatment planning system (TPS, Prowess, version 3.02, SSGI, USA). The radiation oncologist outlined the planning target volume (PTV) on each transverse image, to which the prescribed D90 (the doses delivered to 90% of the target volume) was prescribed. The PTV included the gross tumor volume (GTV) with a 0.5-1 cm margin. A radiation dose of 90-160 Gy was prescribed to the PTV and calculated through computerized treatment planning system.

We had previously reported the implant technique used in this study [14]. Seed implantation was performed in all the 14 patients under local anesthesia in the CT room. After determining the target volume, 18-gauge interstitial needles were inserted into the tumor through the skin surface with PTV under CT supervision. Most of the 18-gauge needles were placed 1.0 cm apart in a parallel array in the PTV. Precautions were taken to avoid puncturing the large vascular and neural structures and the bronchus. The median number of needles is 14 (range, 5-42). After placing the needles, ^125^I seeds (Model 6711; Beijing Atom and High Technique Industries Inc., Beijing) were implanted using a Mick applicator; the seeds were implanted 1.0 cm apart. All the patients received perioperative prophylactic antibiotics.

**Figure 1:**
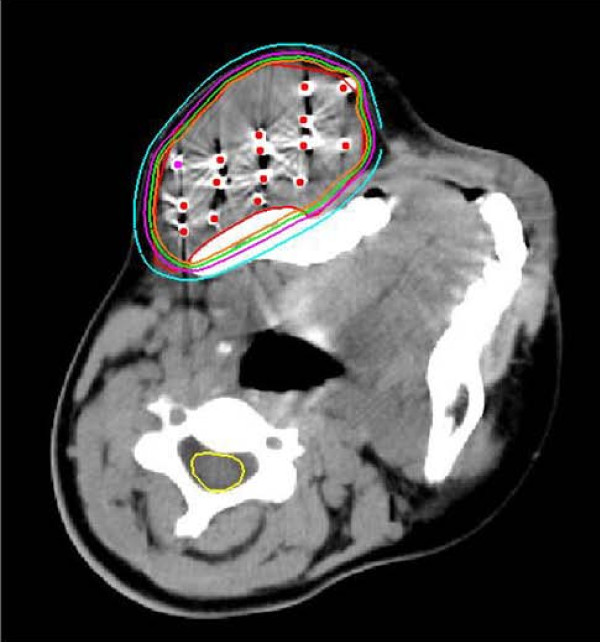
**The isodose curve after seed implantation from CT scan**. The inner red cure represents GTV. The ellipses are iso-dose lines of 160, 140, 120, 90 Gy from inside, respectively.

**Figure 2:**
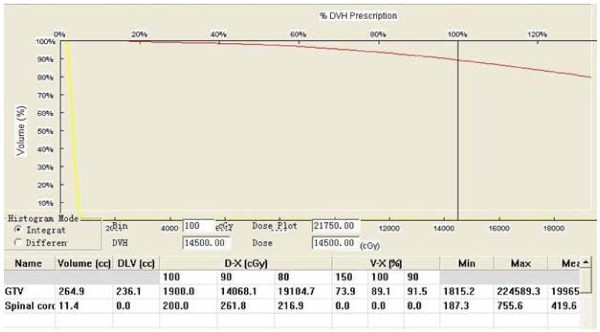
**The Dose volume histograms of GTV and spinal cord after seed implantation**.

Postoperative dosimetric measurements were routinely obtained for all the patients. The implant dosimetry was determined using three-dimensional seed identification, and axial CT images (slice thickness, 5 mm) of the implanted area were obtained immediately or 24 h after seed implantation. The CT-derived postimplant target volumes were defined to encompass the GTV with a 0.5-1 cm margin. The ^125^I seeds were identified on the CT images by using a combination of manual selection and an automated redundancy check feature available on the Prowess treatment planning system. We generated isodose curves for each slice (Fig. 1) and dose-volume histograms of the target (Fig. 2). The actuarial median number of the implanted ^125^I seeds was 48 (range, 21-158). The specific activity of ^125^I seeds ranged from 0.40 to 0.80 mCi/seed (median, 0.65 mCi). The total activity of the implanted seeds ranged from 8.8 to 113.6 mCi (median, 24.9 mCi). The evaluation of post plan shown the actuarial D90 ranged from 90 to 218 Gy (median, 157.5 Gy). The seed implanted volume ranged from 9.1 to 290.4 cm^3 ^(median, 32 cm^3^).

Tumor response was first evaluated at 4 weeks after implantation. Subsequent evaluations were performed at 2-3- month interval for the next 2 years and 6-month interval, thereafter. The disease status was assessed by physical examinations, liver function tests, and complete blood and platelet counts. Disease progression was determined by means of imaging studies, including CT scans and ultrasonography. The follow-up time was calculated from the date of seed implantation. The median follow-up period was 13 months (range, 3-60 months).

The complications were scored using the Radiation Therapy Oncology Group (RTOG)/European Organization for Research and Treatment of Cancer (EORCT) late radiation morbidity score [16].

The survival time was calculated from the date of implantation to the last date of follow-up or date of death. In these calculations, deaths due to any reason were scored as events. Local control was defined as the lack of tumor progression either in or adjacent to the implanted volume. Tumor responses were assessed using CT and ultrasound according to the World Health Organization (WHO) criteria [17]. The overall local control and survival times were determined using the Kaplan-Meier method by using SPSS 10.0 for Windows (SPSS, Chicago, IL).

## Results

### Local control

**Figure 3:**
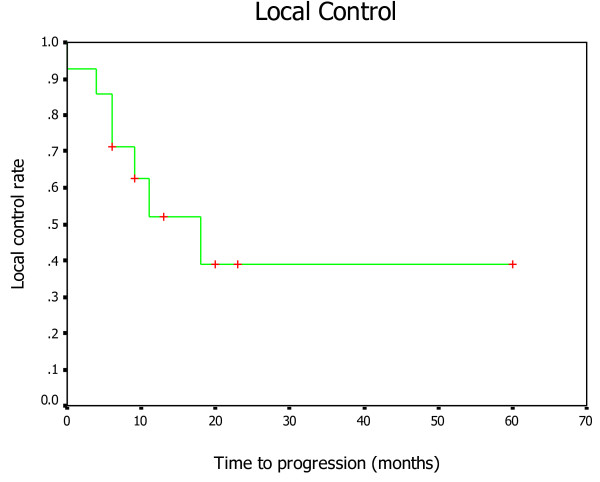
**Kaplan-Meier estimates showing local control for all the patients after ^125^I seed implantation**.

The follow-up period ranged from 3 to 60 months (median, 12 months). The median local control was 18 months (95% CI, 6.1-29.9 months), and the 1-, 2-, 3- and 5-year local controls were 52%, 39%, 39%, and 39%, respectively. Of all patients, 28.6% (4/14) died of local recurrence, 7.1% (1/14) died of metastases, and 1 died of hepatocirrhosis 20 months after seed implantation and 8 still survive to the date of this analysis (Fig. 3).

### Survival

**Figure 4:**
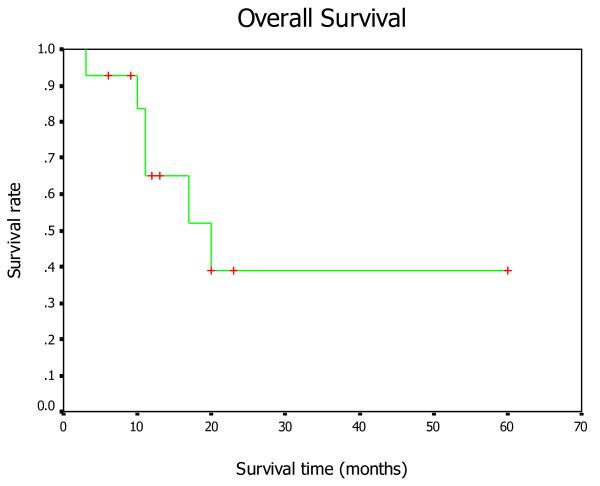
**Kaplan-Meier estimates showing overall survival for all the patients after ^125^I seed implantation**.

The median survival time was 20 months (95% CI, 8.7-31.3 months), and the 1-, 2-, 3- and 5-year survival rates were 65%, 39%, 39%, and 39%, respectively (Fig. 4).

### Complications

One patient had grade one skin reaction, one experienced grade 1 mucosal reaction, and one developed ulceration with tumor progression after 11 months. We did not observe blood vessel damage and neuropathy in the patients.

## Discussion

The treatment of patients with recurrent head and neck cancer in a previously irradiated area is particularly challenging for both surgery and EBRT. In such circumstances, redelivery of EBRT is not possible because of the high radiation dosage previously applied and the tolerance of adjacent normal tissue. However, local control rates of up to 50% and a 5-year survival rate of 20% have been reported after redelivery of EBRT [18-20]. The use of re-irradiation combined with chemotherapy for recurrent head and neck carcinomas, after previous full-dose radiotherapy, has shown encouraging median survivals [21-23]. However, this approach has sometimes met some trouble for patients who have ever received EBRT and worry about the normal tissue damages. The re-irradiation possibility is small for patients who have ever received twice EBRT, even IMRT or IGRT.

Surgery combined with intraoperative HDR interstitial brachytherapy has become a modality of salvage treatment for managing patients with locally advanced or recurrent head and neck cancer, with a local control rate of 40-60% and a 5-year survival rate of 14% [24,25]. Salvage surgery or surgery combined with intraoperative HDR is often technically feasible, but its curative potential is low, and local failure rate is ≥40% [26].

Intraoperative pulsed-dose-rate (PDR) brachytherapy is an alternative reirradiation strategy that lowers the risk of severe morbidity [27]. The use of this technique resulted in excellent local control rates of up to 80% with minimal side effects when performed in carefully selected patients [28]. Intraoperation PDR imposed some practical limitations, the tumor size and location of most recurrences inhibited its application. Normal tissue fibrosis after EBRT can impair bimanual palpation of recurrent tumors during the needle implantation, the organ at risk is often in the vicinity of the tumor. The patient treatment usually lasted several days. Radium-226 and iridium-192 have been used for T1 and T2 patients with oral cavity and oropharynx carcinomas and produced excellent control rates and functional results [29-33]. Vikram et al reported 124 patients with advanced recurrent head and neck cancer who were treated using ^125^I implants [34]; 71% of these patients showed complete regression, 18% showed >50% regression, and 11% showed no response. Overall, local control was achieved in 64% of the patients until their deaths. Only 9% of the patients survived for 2 years and 5.5% survived for 5 years. Goffinet et al reported using permanent ^125^I seed implants as a surgical adjuvant in the management of patients with advanced recurrent head and neck cancer; most of the patients in this study had received prior treatment. Management involved a salvage operation combined with permanent ^125^I seed implants, and a local control rate of 70% had been achieved [35]. Park et al reported 35 patients with advanced recurrent squamous cell cancers of the head and neck that were treated with surgical resection followed by adjuvant ^125^I seed implantation. The 5-year disease-free survival rate was 41% [36]. The use of concomitant intraoperative HDR or LDR brachytherapy has been shown to enhance actuarial survival and local control rates, but the occurrence of local complications have also been reported in 11-56% of cases [37]. Martinez et al reported an overall complication rate of 11% [37], and Goffinet et al reported an overall complication rate of approximately 50% [35]. The main complications were skin ulceration and wound breakdown; however, carotid rupture, which is a fatal complication, has also been occasionally reported.

Catheters are inserted during intraoperative HDR or PDR brachytherapy on the basis of preoperative images and intraoperative presentation. Planning of intraoperative HDR or PDR brachytherapy has a number of potential disadvantages: (1) the target volume and shape may change between the time of pre-plan and real time implant insertion; (2) the dose calculation is depended on pre-plan, and did not realized real time and post-plan in operation or after operation. However, image-guided seed permanent implantation overcomes these disadvantages in some selected patients. We observed a very low rate of complications, one patient had grade one skin reaction, one experienced grade one mucosal reaction, and one developed ulceration with tumor progression after 11 months, and no adverse events were attributable to seed implantation itself. One patient had ulceration because of tumor progression after seed implantation. We did not observe the occurrence of bone and soft tissue necrosis, carotid rupture, or other grade 4 or 5 toxicity.

Image-guided HDR brachytherapy for non-small-cell lung cancer and other malignancies has recently been reported by other groups [38-41]. This enables the use of circumscribed high-dose radiotherapy for the tumor target as well as the safety margin. CT-guided ^125^I permanent seed implantation has the following advantages: (1) the implantation technique is supervised real timely on CT scan and can be performed easily under local anesthesia; (2) the possibility of target geographical miss is reduced; (3) radiation dose to the surrounding tissues is minimized due to the sharp dose fall-off outside the implanted volume, thereby lowering the morbidity; and (4) it has a shorter treatment time than re-irradiation or surgery combined with HDR. Krempien et al have reported that frameless image-guided interstitial needle implantation was feasible and accurate for 14 patients with locally recurrent head and neck cancers [42]. The 1- and 2-year local control rates were 78% and 57%, respectively, and the actuarial 1- and 2-year survival rates were 83% and 64%, respectively. Image guidance allows virtual planning and navigated needle implantation, which, in turn, facilitates optimized dose distribution and reduces adjacent tissue damage.

We elaborated this technique for routine use in a large number of recurrent cancer patients by using CT-guidance and routinely obtained a standard postoperative dosimetry for all patients using the 3D-CT dataset. This demonstrated that a good tumor control rate and low complication rate could be achieved using percutaneous CT-guided ^125^I seed permanent implantation while treating recurrent carcinomas [14,15]. Our results showed that the 1-, 2-, 3-, and 5-year control rates were 52%, 39%, 39%, and 39%, respectively, with a median local control of 18 months. The 1-, 2-, 3-, and 5-year actuarial overall survival rates were 65%, 39%, 39%, and 39%, respectively, with a median survival of 20 months.

In conclusion, CT-guided ^125^I seed permanent implantation is an effective salvage modality of radiotherapy for recurrent head and neck carcinoma after previous surgery or EBRT. It eliminates the need for further surgery or EBRT, improves the outcome, and low side effects. Considering the limited number of patients involved in this study, arriving at a definite conclusion requires a large number of patients and long-term follow-up.

## Abbreviations

The abbreviations used are: ^125^I: iodine-125; LDR: low-dose rate; HDR: high-dose rate; SLD: sublethal damage; TPS: treatment planning system; EBRT: external beam radiotherapy; GTV: gross tumor volume; PTV: planning target volume; LR: local recurrence; CR: complete response; PR: partial response; SD: stable disease; PD: progressive disease; TTP: time to progression; OS: overall survival.

## Conflict of interests statement

We disclose to Radiation Oncology the following potential conflicts of interest: this study is supported by the Fund of Capital Medical Development and Research, item NO. 2009-2024.

## Authors' contributions

YLJ, NM, AQ and PJ participated in the data collection and performed the statistical analysis, HSY and CL carried out the needle penetration, RJY carried the dose calculation of seed implantation, JJW participated in the design of the study and seed implantation. All authors read and approved the final manuscript.

## References

[B1] Jemal A Siegel R Ward E Murray T Xu J Smigal C Thun MJ Cancer Statistics, 2006 CA Cancer J Clin 2006 56 106 130 10.3322/canjclin.56.2.106 16514137

[B2] Marcial VA Pajak TF Kramer S Yupchong L Stetz J Radiation Oncology Group (RTOG) studies in head and neck cancer Semin Oncol 1998 15 39 60 3278390

[B3] Ridge JA Squamous cancer of the head and neck: Surgical treatment of local and regional recurrence Semin Oncol 1993 20 419 429 7692603

[B4] Wang CC Re-irradiation of recurrent nasopharyngeal carcinoma-treatment techniques and results Int J Radiat Oncol Biol Phys 1987 13 953 956 3597157 10.1016/0360-3016(87)90030-7

[B5] Harrison LB Application of brachytherapy in head and neck cancer Semi Surg Oncol 1997 13 177 184 10.1002/(SICI)1098-2388(199705/06)13:3<177::AID-SSU4>3.0.CO;2-4 9143055

[B6] Goffinet D Fee WE Jr Wells J Austin-Seymour M Clarke D Mariscal JM ^192^Ir pharyngoepiglottic fold interstitial implants. The key to successful treatment of bare tongue carcinoma by radiation therapy Cancer 1985 55 941 948 10.1002/1097-0142(19850301)55:5<941::AID-CNCR2820550505>3.0.CO;2-G 3967201

[B7] Hilaris BS Lewis JS Henschke MK Therapy of recurrent cancer of the nasopharynx Arch Otolaryngol 1968 87 506 510 4171548 10.1001/archotol.1968.00760060508012

[B8] Wang CC Burse K Gitterman M A simple afterloading applicator or intracavitary irradiation of carcinoma of the nasopharynx Radiology 1975 115 737 738 1129494 10.1148/15.3.737

[B9] Ash D Interstitial therapy Acta Radiol 1986 16 369 393

[B10] Syed AMN Puthawala A Afterloading interstitial implants in head and neck cancer Arch Otolaryngol 1980 106 541 546 6996657 10.1001/archotol.1980.00790330021008

[B11] Lee DJ Liberman FZ Park RI Zinreich ES Intraoperative I-125 seed implantation for extensive recurrent head and neck carcinomas Radiology 1991 178 879 882 1994436 10.1148/radiology.178.3.1994436

[B12] Ricke J Wust P Stohlmann A Beck A Cho CH Pech M Wieners G CT-guided brachytherapy. A novel percutaneous technique for interstitial ablation of liver malignancies Strahlenther Onkol 2004 180 274 280 10.1007/s00066-004-1179-4 15127157

[B13] Ricke J Wust P Stohlmann A Beck A Cho CH Seidensticker M Wieners G Spors B Werk M Rosner C CT-guided brachytherapy of liver malignancies alone or in combination with thermal ablation: phase I-II results of a novel technique Int J Radiat Oncol Biol Phys 2004 58 1496 1505 10.1016/j.ijrobp.2003.09.024 15050329

[B14] Jun JW Hui ShuY Ji NL Wei JJ Yu LJ Su QT Interstitial permanent implantation of ^125^I seeds as salvage therapy for re-recurrent rectal carcinoma Int J Colorectum Dis 2009 24 391 399 10.1007/s00384-008-0628-4 19084969

[B15] Jun JW Hui ShuY Qing JM Xiao GL Hao W Yu LJ Su QT Rui JY Interstitial ^125^I seeds implantation to treat spinal metastatic and primary paraspinal malignancies Med Oncol 2009 10.1007/s12032-009-9212-1 10.1007/s12032-009-9212-1 19360383

[B16] Cox JD Stetz J Pajak TF The toxicity criteria of the Radiation Therapy Oncology for Research and Treatment of Cancer (EORTC) Int J Radiat Oncol Biol Phys 1995 31 1341 1346 7713792 10.1016/0360-3016(95)00060-C

[B17] Miller AB Hoogstraten B Staquet M Winkler A Reporting results of cancer treatment Cancer 1981 47 207 214 10.1002/1097-0142(19810101)47:1<207::AID-CNCR2820470134>3.0.CO;2-6 7459811

[B18] Emami B Bignardi M Devineni VR Spector GJ Hederman MA Re-irradiation of head and neck cancer Laryngoscope 1987 97 85 88 10.1288/00005537-198701000-00016 3796178

[B19] Kennedy JT Krause CJ Loevy S The importance of tumor attachment to the carotid artery Arch Otolaryngol Head Neck Surg 1977 103 70 73 10.1001/archotol.1977.00780190050002 836232

[B20] Langois D Eschwege F Kramer A Richard JM Re-irradiation of head and neck cancers Radiother Oncol 1985 3 27 33 10.1016/S0167-8140(85)80006-2 3975439

[B21] Pomp J Levendage PC van Putten WLJ Re-irradiation with recurrent tumors in the head and neck Am J Clin Oncol 1988 11 543 549 10.1097/00000421-198810000-00007 3177256

[B22] Spencer SA Harris J Wheeler RH Machtay M Schultz C Spanos W Rotman M Spanos W Rotman M RTOG 96-10: Re-irradiation with concurrent hydroxyurea and 5-flurouracil in patients with squamous cell cancer of the head and neck Int J Radiat Oncol Biol Phys 2001 51 1299 1304 11728690 10.1016/s0360-3016(01)01745-x

[B23] Stevens KR Britsch A Moss WT High-dose re-irradiation of head and neck cancer with curative intent Int J Radiat Oncol Biol Phys 1994 29 687 698 8040014 10.1016/0360-3016(94)90555-x

[B24] Emami B Marks JE Re-irradiation of recurrent carcinoma of the head and neck by afterloading interstitial 192Ir implant Laryngoscope 1983 93 1345 1347 6621236 10.1002/lary.1983.93.10.1345

[B25] Mazeron JJ Langlolis D Glaubinger D Huart J Martin M Raynal M Calitchi E Ganem G Salvage irradiation of oropharyngeal cancers using iridium 192 wire implants: 5-year results of 70 cases Int J Radiat Oncol Biol Phys 1987 13 957 962 3597158 10.1016/0360-3016(87)90031-9

[B26] Puthawala AA Syed AMN Interstitial re-irradiation for recurrent and/or persistent head and neck cancers Int J Radiat Oncol Biol Phys 1987 13 1113 1114 3597154 10.1016/0360-3016(87)90052-6

[B27] Geiger M Strnad V Lotter M Sauer R Pulsed-dose rate brachytherapy with concomitant chemotherapy and interstitial hyperthermia in patients with recurrent head-and -neck cancer Brachytherapy 2002 1 149 153 10.1016/S1538-4721(02)00056-9 15090278

[B28] Strnad V Geiger M Lotter M Sauer R The role of pulsed-dose-rate brachytherapy in previously irradiated head-and-neck cancer Brachytherapy 2003 2 158 163 10.1016/S1538-4721(03)00132-6 15062138

[B29] Senan S Levendage PC Brachytherapy for recurrent head and neck cancer Hematol Oncol Clin North Am 1999 13 531 542 10.1016/S0889-8588(05)70073-3 10432427

[B30] Decroix Y Ghossein NA Experience of the Curie Institute in treatment of cancer of the mobile tongue Cancer 1981 47 496 502 10.1002/1097-0142(19810201)47:3<496::AID-CNCR2820470312>3.0.CO;2-Q 7226001

[B31] Gilbert EH Goffinet DR Ragshaw WA Carcinoma of the oral tongue and floor of mouth: Fifteen years experience with linear accelerator therapy Cancer 1975 35 1517 1524 10.1002/1097-0142(197506)35:6<1517::AID-CNCR2820350607>3.0.CO;2-3 807312

[B32] Shasha D Harrison LB Chiu-Tsao ST The role of brachytherapy in head and neck cancer Semin Radiat Oncol 1998 8 270 281 10.1016/S1053-4296(98)80025-8 9873105

[B33] Fee WE Goffinet DR Paryani S Goode RL Levine PA Hopp ML Intraoperative Iodine-125 implants Arch Otolaryngol 1983 109 727 730 6639439 10.1001/archotol.1983.00800250021005

[B34] Vikram B Hilaris BS Anderson L Strong EW Permanent iodine-125 implants in head and neck cancer Cancer 1983 51 1310 1314 10.1002/1097-0142(19830401)51:7<1310::AID-CNCR2820510722>3.0.CO;2-I 6186353

[B35] Goffinet DR Martinez A Fee WE Jr I-125 vicry suture implants as a surgical adjuvant in cancer of the head and neck Int J Radiat Oncol Biol Phys 1985 11 399 402 3972656 10.1016/0360-3016(85)90164-6

[B36] Park RI Liberman FZ Lee DJ Goldsmith MM Price J Iodine-125 seed implantation as an adjuvant to surgery in advanced recurrent squamous cell cancer of the head and neck Laryngoscope 1991 101 405 410 10.1288/00005537-199104000-00012 1895857

[B37] Martinez A Goffinet DR Fee W Goode R Cox RS Iodine-125 implants as an adjuvant to surgery and external beam radiotherapy in the management of locally advanced head and neck cancer Cancer 1983 51 973 979 10.1002/1097-0142(19830315)51:6<973::AID-CNCR2820510602>3.0.CO;2-T 6821872

[B38] Ricke J Wust P Wieners G Hengst S Pech M Hanninen EL Felix R CT-guided interstitial single fraction HDR brachytherapy of lung tumors: Phase I results of a novel technique Chest 2005 127 2237 2242 10.1378/chest.127.6.2237 15947343

[B39] Krempien RC Grehn C Haag C Straulino A Hensley EW Kotrikova B Hofele Ch Debus J Harms W Feasibility report for retreatment of locally recurrent head-and neck cancers by combined brachytherapy using frameless image-guided 3 D interstitial brachytherapy Brachytherapy 2005 4 154 162 10.1016/j.brachy.2005.02.001 15893270

[B40] Harms W Krempien R Grehn C Hensley F Debus J Becker HD Electromagnetically navigated brachytherapy as a new treatment option for peripheral pulmonary tumors Strahlenther Onkol 2006 182 108 111 10.1007/s00066-006-1503-2 16447018

[B41] Griffin PC Amin PA Hughes P Levine AM Sewchand WW Salazar OM Pelvic mass: CT-guided interstitial catheter implantation with high-dose-rate remote afterloader Radiology 1994 191 581 583 8153347 10.1148/radiology.191.2.8153347

[B42] Kolotas C Baltas D Zamboglu N CT-based interstitial HDR brachytherapy Strahlenther Onkol 1999 175 419 427 10.1007/s000660050031 10518974

